# Epigenetic regulation of inflammation in periodontitis: cellular mechanisms and therapeutic potential

**DOI:** 10.1186/s13148-020-00982-7

**Published:** 2020-11-30

**Authors:** Krzysztof T. Jurdziński, Jan Potempa, Aleksander M. Grabiec

**Affiliations:** 1 Department of Microbiology, Faculty of Biochemistry, Biophysics and Biotechnology, Jagiellonian University, Kraków, Poland; 2 Department of Oral Immunology and Infectious Diseases, University of Louisville School of Dentistry, Louisville, KY USA

**Keywords:** Periodontitis, Epigenetics, DNA methylation, Histone deacetylase, Bromodomain, Inflammation, *Porphyromonas gingivalis*

## Abstract

Epigenetic mechanisms, namely DNA and histone modifications, are critical regulators of immunity and inflammation which have emerged as potential targets for immunomodulating therapies. The prevalence and significant morbidity of periodontitis, in combination with accumulating evidence that genetic, environmental and lifestyle factors cannot fully explain the susceptibility of individuals to disease development, have driven interest in epigenetic regulation as an important factor in periodontitis pathogenesis. Aberrant promoter methylation profiles of genes involved in inflammatory activation, including *TLR2*, *PTGS2*, *IFNG*, *IL6*, *IL8,* and *TNF*, have been observed in the gingival tissue, peripheral blood or buccal mucosa from patients with periodontitis, correlating with changes in expression and disease severity. The expression of enzymes that regulate histone acetylation, in particular histone deacetylases (HDACs), is also dysregulated in periodontitis-affected gingival tissue. Infection of gingival epithelial cells, gingival fibroblasts and periodontal ligament cells with the oral pathogens *Porphyromonas gingivalis* or *Treponema denticola* induces alterations in expression and activity of chromatin-modifying enzymes, as well as site-specific and global changes in DNA methylation profiles and in histone acetylation and methylation marks. These epigenetic changes are associated with excessive production of inflammatory cytokines, chemokines, and matrix-degrading enzymes that can be suppressed by small molecule inhibitors of HDACs (HDACi) or DNA methyltransferases. HDACi and inhibitors of bromodomain-containing BET proteins ameliorate inflammation, osteoclastogenesis, and alveolar bone resorption in animal models of periodontitis, suggesting their clinical potential as host modulation therapeutic agents. However, broader application of epigenomic methods will be required to create a comprehensive map of epigenetic changes in periodontitis. The integration of functional studies with global analyses of the epigenetic landscape will provide critical information on the therapeutic and diagnostic potential of epigenetics in periodontal disease.

## Introduction

Periodontitis is an inflammatory disease initiated and sustained by oral microbial biofilm (dental plaque) [1]. While keystone pathogens, such as *Porphyromonas gingivalis*, *Tannerella forsythia,* and *Treponema denticola*, play crucial roles in disease development and progression, it is now commonly accepted that periodontitis is caused by dysbiosis rather than specific periodontal pathogen(s) [2]. In periodontitis-affected gingival tissue, infiltrating immune cells and resident gingival cells, in particular gingival epithelial cells (GECs) and fibroblasts, produce excessive amounts of cytokines, chemokines, and matrix-degrading enzymes in a futile attempt to eliminate periodontal pathogens. Instead, chronic production of these mediators drives inflammatory tissue breakdown, which provides nutrients for inflammophilic bacteria. As a consequence, non-resolving inflammation both sustains dysbiotic microbiota and is the main cause of periodontitis-related tissue damage [3]. The rapidly growing body of evidence indicates that apart from the inevitable tooth loss, untreated periodontitis is strongly associated with increased risk of developing a number of systemic diseases, such as rheumatoid arthritis [4], Alzheimer’s disease [5], cardiovascular disease, diabetes, and cancer [6].

Historically, the most common forms of non-necrotizing periodontitis have been divided into two pathophysiologically distinct forms of disease: chronic periodontitis, characterized by late-onset and slow progression, and less common aggressive periodontitis, characterized by early-onset and rapid progression [7]. While aggressive periodontitis is strongly associated with genetic risk factors, the importance of the genetic component is much less evident in patients with chronic disease [8]. Although a new classification system for periodontitis has been recently developed [7], a vast majority of the epigenetic studies discussed in this review have been conducted on patient cohorts assembled before the introduction of these classification criteria and used the distinction between chronic and aggressive disease. Here, we will discuss studies performed on patients with the chronic form of the disease, referring to them as *periodontitis patients*, as well as reports describing groups of young individuals with above-average speed of disease progression, calling them *aggressive periodontitis patients*.

Due to the significant morbidity of periodontitis and associated diseases, there is a pressing need for developing novel therapeutic approaches to support the currently used strategies that focus on limiting the bacterial challenge (scaling and root planning or root surface debridement). Because of that, there is a growing interest in a new therapeutic approach, called host modulation therapy. This strategy is based on the idea that conventional periodontal treatment is supported by therapeutic agents that ameliorate destructive consequences of the host inflammatory response [1]. The emergence of this concept coincided with the identification of essential roles of epigenetic mechanisms in immunity and inflammation and the anti-inflammatory potential of epigenetic drugs [9, 10]. This, in combination with evidence that genetic, environmental, and lifestyle factors cannot fully explain the susceptibility of individuals to disease development [11], has driven substantial interest in epigenetic regulation as an important factor in periodontitis pathogenesis and a potential target for host modulation therapy.

## Epigenetic mechanisms

The meaning of the term “epigenetics” varies across scientific literature [12]. We will use this term in accordance with one of the unifying definitions, proposed by Bird: “*the structural adaptation of chromosomal regions so as to register, signal, or perpetuate altered activity states,*” [13] focusing on potentially heritable changes not involving alterations in DNA sequences [14]. Among these changes, DNA methylation and posttranslational modifications (PTMs) of histones are the best characterized.

**Fig. 1:**
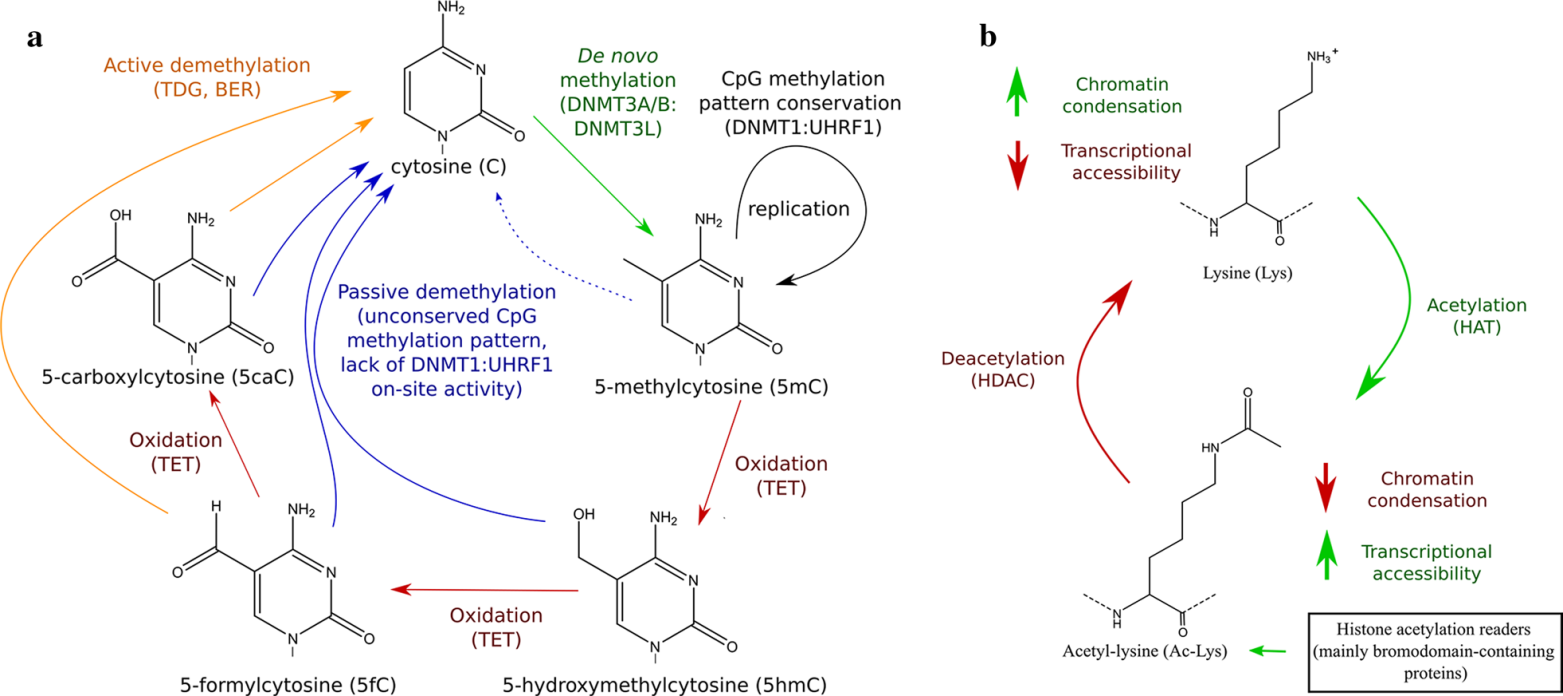
Schematic representation of the biochemical processes involved in DNA methylation and histone acetylation. **a** Unmethylated cytosine at the CpG site can be methylated de novo by DNMT3A or DNMT3B in complex with DNMT3L, the latter lacking methyltransferase activity. The DNMT1:UHRF1 complex is responsible for the conservation of the CpG methylation pattern on the complementary DNA strand after replication. TET enzymes catalyze the formation of oxidized 5mC derivatives, which are no longer recognized by the DNMT1:UHRF1 complex (passive demethylation). Additionally, 5fC and 5caC can be recognized and excised by TDG, leading to replacement with unmodified cytosine through BER (active demethylation). **b** N-terminal lysine residues on histone tails are acetylated by HATs, which leads to neutralization of their positive charge, relaxation of chromatin structure, and increased transcriptional accessibility of gene promoters. Bromodomain-containing proteins recognize specific acetyl-lysine containing sequences within the histone, promoting the formation of acetylation-dependent transcriptional complexes. Acetylated lysine residues can be deacetylated by HDACs. *BER* base-excision repair, *DNMT* DNA methyltransferase, *HAT* histone acetyltransferase, *HDAC* histone deacetylase, *TET* ten-eleven translocation, *TDG* thymine DNA glycosylase, *UHRF1* Ubiquitin-like, containing PHD and RING finger domains-1

Methylation of cytosine is the most common modification of DNA that occurs at CpG dinucleotides (cytosine followed by guanine). It typically causes chromatin condensation and disruption of interactions between DNA and transcription factors, which are associated with transcriptional repression [15]. In addition to 5-methylcytosine (5mC) residues, stable oxidized 5mC derivatives, such as 5-hydroxymethylcytosine (5hmC), are also commonly found throughout the genomic DNA. They are intermediates of DNA demethylation [16] that play independent roles in functional chromatin organization [17]. DNA methylation marks are established, recognized, and deleted in a site-specific manner. Cytosine residues are methylated by DNA methyltransferases (DNMTs) and can undergo passive or active demethylation (Fig. 1a). DNMT1 in a complex with UHRF1 (ubiquitin like with PHD and ring finger domains 1) is responsible for conservation of CpG methylation after replication, whereas DNMT3a or DNMT3b in a complex with DNMT3L catalyze de novo methylation [15]. Both passive and active demethylation can involve oxidation of 5mC and its oxidized derivatives by TET (ten-eleven translocation) enzymes [16]. Active demethylation occurs through excision of 5-formylcytosine (5fC) or 5-carboxylcytosine (5caC) by thymine DNA glycosylase (TDG) and base-excision repair (BER) [16]. In contrast, DNA undergoes passive demethylation in the course of DNA replication when DNMT1 is lacking or inhibited, or when the DNMT1:UHRF1 complex does not recognize the CpG site to be methylated as a result of 5mC oxidation [16].

Histones undergo a plethora of different PTMs, including acetylation, methylation, phosphorylation, citrullination, and ubiquitylation. They have various, often context-dependent effects on transcriptional activity and chromatin structure [15]. As it was unclear whether histone PTMs are heritable, their status as epigenetic marks has been elusive. Still, recent reports provided clear evidence that some of these modifications are indeed mitotically stable in a locus-specific manner and this inheritance is biologically relevant [18, 19].

Amongst histone PTMs, acetylation is the best characterized in terms of mechanisms of regulation and effects on transcription (Fig. 1b). Histone acetyltransferases (HATs) acetylate lysine residues on histones while histone deacetylases (HDACs) remove acetyl groups [15]. Histone acetylation is strongly associated with increased transcriptional activity, though distinct acetylation sites are involved in this process at different levels. Acetylation of lysines on H3 globular domains (in particular histone 3 lysine 56 [H3K56]) and H4K16 disrupt electrostatic interactions within and between the nucleosomes, promoting relaxation of the chromatin structure [20]. Acetylated lysines on H3 and H4 tails act as binding sites for specific bromodomain-containing reader proteins, such as bromodomain and extra-terminal (BET) proteins, which regulate transcription-related processes [21]. However, recent evidence indicates that the interplay between acetylation and deacetylation in transcriptional regulation is more complex. Genome-wide mapping of HAT and HDACs revealed their unique functions at active and inactive genes, demonstrating that dynamic cycles of acetylation and deacetylation poise inactive genes for future activation [22]. Importantly, despite its essential role in transcriptional activation, histone acetylation alone is not sufficient for the induction of gene expression. This is exemplified by studies of HDAC inhibitors (HDACi). Although these compounds induce global histone hyperacetylation, they affect the expression of a relatively small proportion of the transcribed genes, many of which are downregulated [23]. Similarly, lysine methylation, which is established by histone methyltransferases (HMTs) and removed by histone demethylases, affects chromatin structure and gene expression in a context-dependent manner [15]. For example, trimethylation marks at H3K27 and H3K9 are associated with repressed genes, whereas H3K4Me3 and H3K36Me3 have been linked to transcriptional activation [24].

It should be noted that regulation of gene expression by non-coding RNAs, in particular by micro-RNAs, is classified as an epigenetic mechanism by some researchers. However, the contributions of non-coding RNAs to periodontitis pathogenesis have been reviewed elsewhere [25, 26] and will not be covered in this review. Similarly, the involvement of epigenetic regulation in periodontal disease has been thoroughly reviewed in the context of bone metabolism [27] and potential links with oncogenesis [28], so we will not discuss these aspects extensively here. In this review, we will focus predominantly on molecular and cellular processes affected by epigenetic mechanisms and their clinical implications.

## Posttranslational histone modifications and chromatin-modifying enzymes in periodontitis

### Alterations in expression and function of chromatin-modifying enzymes

In many chronic inflammatory diseases, alterations in expression and function of proteins that regulate posttranslational histone modifications are associated with pathology. Similarly, several bacterial pathogens manipulate histone acetylation in host cells to facilitate survival and evade the immune response [23]. Not surprisingly, changes in the expression patterns of proteins involved in chromatin remodeling have been identified in the inflamed gingival tissue and cells of the periodontium exposed to oral pathogens. Global HDAC expression profiling in the gingival tissue from patients with periodontitis and healthy controls produced conflicting data. Ateia et al. [29] reported reduced expression of several classes of chromatin-modifying enzymes, including class II HDACs in periodontitis-affected gingival tissue, though the differences were not verified at the protein level. Conversely, an independent study identified elevated transcript levels of several HDAC family members, including HDAC1, HDAC5, HDAC8, and HDAC9, in periodontitis patients compared to healthy individuals, though only the increased HDAC1 expression was confirmed at the protein level in immunohistochemical studies of the gingival tissue [30]. Due to the limited sample sizes and the lack of detailed clinical characteristics of the analyzed patients, it is difficult to resolve the apparent discrepancies between these studies. Previous evidence from other pathologies, in particular rheumatoid arthritis (RA), suggests the importance of conducting more extensive analyses of the equilibrium between HAT and HDAC activity, global and loci-specific histone acetylation as well as relationships with clinical and inflammatory disease parameters [31, 32]. In this regard, it is noteworthy that elevated HDAC1 expression in the gingival tissue was associated with cells expressing tumor necrosis factor (TNF) [30]. Similar positive correlations between expression of TNF and HDAC1, as well as other class I HDACs, have been reported in the synovial tissue from RA patients [33, 34]. This observation suggests that increased levels of HDAC1 in periodontitis may be related to the ongoing inflammatory process rather than interaction with oral bacteria.

Studies of cells isolated from periodontal tissues revealed that HDAC expression is dynamically regulated by oral pathogens. Reduced expression of HDAC1 and HDAC2 was observed in GECs upon infection with *P. gingivalis* [35], whereas *T. denticola* challenge downregulated transcript levels of several class II HDAC family members, including HDAC4, HDAC6, and HDAC10 in periodontal ligament (PDL) cells [29]. The reported downregulation of HDACs by oral pathogens favors histone hyperacetylation and, indeed, oral pathogens and lipopolysaccharide (LPS) have been shown to increase global H3K9 acetylation in GECs and the gingival tissue in murine periodontitis [36]. However, HDAC expression was not evaluated in this study, and elevated H3K9 acetylation was attributed to activation of the HAT p300/CBP [36].

It is well established that dynamic regulation of activating and repressive histone methylation marks is essential for coordination of macrophage responses to LPS [37]. Accumulating evidence indicates that oral pathogens can induce rapid changes in histone methylation also in other cell types. LPS stimulation of PDL cells leads to the accumulation of the activating H3K4Me3 marks and reduction of repressive H3K27 trimethylation on inflammatory gene promoters [38, 39]. Histone demethylase JMJD3 (KDM6B), which catalyzes H3K27 demethylation, is recruited to *IL6* and *IL12B* promoters and promotes gene transcription [38]. Similarly, the H3K4 methyltransferase SETD1B is upregulated in LPS-stimulated PDL cells and accumulates on the promoters of *IL6* and *IL1B* genes [39]. Dynamic regulation of H3K27 as well as H3K4 trimethylation marks at the promoters of matrix-related and osteogenic genes (*COL1A1*, *COL3A1*, and *RUNX2*) and other inflammatory mediators (*CCL5*, *IL1B*) have also been observed in PDL cells following LPS stimulation [40]. These changes favor a transcriptional program that suppresses PDL osteogenic differentiation [40]. In line with observations in isolated cell populations, unique histone methylation signatures have been observed in experimental periodontitis in mice [41]. Among them, the repressive H3K27Me3 mark appears to be most dynamically regulated under inflammatory conditions. H3K27 trimethylation is enriched on the promoters of genes involved in extracellular matrix (ECM) turnover and reduced on cytokine, chemokine, and defensin gene promoters in samples from periodontitis-affected mice [41]. However, changes in histone methylation marks on the promoters of genes regulating early signaling events that are critical for Toll-like receptor (TLR)- and cytokine-induced responses have not been evaluated in the context of periodontal inflammation.

### Modulation of chromatin-modifying enzymes by bacterial metabolic products

In periodontitis, anaerobic bacteria modulate HDAC function also indirectly through their fermentation products, such as short-chain fatty acids (SCFAs), some of which display HDAC inhibitory activity. Among them, butyric acid and propionic acid are present in the gingival crevices of patients with severe periodontitis at millimolar concentrations that correlate with inflammatory and clinical parameters of disease activity [42, 43]. To date, the potential pathogenic consequences of the HDACi activity of SCFAs produced by oral bacteria have only been studied in the context of viral infections. SCFAs produced by *P. gingivalis* induce reactivation of latent viruses, including Epstein-Barr virus [44, 45] and Kaposi’s Sarcoma-associated herpesvirus [46], through stimulation of lytic replication. This process is associated with increased H4K12 acetylation, which promotes transactivation of the viral chromatin [46], suggesting that manipulation of HDAC activity by SCFAs may be responsible for the clinical progression of viral infections. Interestingly, SCFA effects on host epigenetic mechanisms may be broader than HDAC inhibition as they have also been shown to decrease repressive histone trimethylation marks H3K27Me3 and H3K9Me3 by downregulating histone methyltransferases EZH2 and SUV39H1 [46].

SCFAs released by oral pathogens may contribute to the pathogenesis of periodontitis also through their effects on structural cells of the gingival tissue. Butyric acid inhibits proliferation and/or induces apoptosis of GECs and fibroblasts in vitro [47, 48]. High concentrations of butyric acid also modulate the RANKL/OPG ratio in osteoblasts, favoring bone resorption, though this in vitro effect is highly dependent on SCFA concentration and time of cell exposure [49]. In contrast, a recent study demonstrated that SCFAs protect against inflammation-mediated bone loss by suppressing osteoclast differentiation without affecting bone formation [50]. Additional studies are therefore needed to fully characterize the role of butyric acid, and other bacterial metabolites, in bone homeostasis in periodontitis.

### Suppression of inflammation by small molecule inhibitors of chromatin-modifying enzymes

It is now well established that small molecule inhibitors of chromatin-modifying enzymes may affect cellular processes that are dysregulated in pathologies associated with aberrant activation of the immune system, disruption of connective tissue homeostasis, and uncontrolled bone resorption [9, 51]. Among them, HDACi have received much interest due to their anti-inflammatory and anti-osteoclastogenic properties [52]. We have recently demonstrated that treatment of gingival fibroblasts with the pan-HDACi suberoylanilide hydroxamic acid (SAHA) and ITF2357 (givinostat) can suppress *P. gingivalis*-induced expression of a broad range of inflammatory mediators involved in periodontitis pathogenesis, including chemokines (*CCL2*, *CCL5,* and *CXCL10*), matrix metalloproteinases (*MMP1*, *MMP3*), and components of the prostaglandin E2 synthesis pathway (*PTGS2*) [53]. HDACi exert similar anti-inflammatory effects on cells infected with *F. nucleatum* or stimulated with TNF without affecting gingival fibroblast viability or susceptibility to bacterial invasion [53]. Application of a panel of HDACi with distinct selectivity profiles revealed that HDAC3 inhibition is sufficient for suppression of inflammatory genes in gingival fibroblasts [53]. These findings are consistent with previous observations in synovial fibroblasts [54] and macrophages [55]. Similarly, in PDL cells, HDACi suppress LPS-induced TNF and IL-1β expression and production of reactive oxygen species [56], and *T. denticola*-induced MMP2 activation [29].

In PDL cells, treatment with HDACi not only reduces inflammatory activation but also results in upregulation of osteoblast markers, elevated levels of the transcription factor RUNX2, increased alkaline phosphatase activity, and deposition of mineralized nodules, which are hallmarks of osteogenic differentiation [56, 57], raising the possibility that these compounds may facilitate alveolar bone regeneration. Interestingly, HDAC expression profiling in the course of PDL cell differentiation into osteoblasts revealed significant downregulation of HDAC3, and this process was accelerated by HDACi [57]. Together, these findings provide initial evidence suggesting that inhibition of HDAC activity, in particular selective targeting of HDAC3, may be clinically beneficial in suppressing inflammation and promoting tissue regeneration in periodontitis (Table 1). HDACs may thus represent a new class of targets for host modulation therapies.

**Table 1:** Effects of small-molecule inhibitors of epigenetic regulators in cellular and animal models of periodontitis

Epigenetic target	Compound(s)	Effect
HDACs	SAHA, ITF2357	Suppression of *P. gingivalis*- and cytokine-induced CCL2, CCL5, CXCL10, MMP1, MMP3 and PTGS expression in gingival fibroblasts [53]
	TSA, butyrate	Upregulation of hBD2, IL-8 and CCL20 in GECs infected with *P. gingivalis* or *F. nucleatum* [35]
	butyrate	Suppression of LPS-induced TNF and IL-1β expression and ROS production in PDL cells [56]
	TSA, apicidin	Suppression of *T. denticola*-induced MMP2 activation in PDL cells [29]
	TSA, butyrate	Upregulation of osteoblast markers and induction of osteogenic differentiation of PDL cells [56, 57]
	1179.4b	Reduction of alveolar bone destruction in experimental periodontitis in mice [69]
	TSA	Reduction of inflammation and increased alveolar bone volume in experimental periodontitis in rats [68]
BET proteins	I-BET151, JQ1	Suppression of inflammatory mediator production by GECs and gingival fibroblasts [65]
	JQ1	Amelioration of inflammation and alveolar bone resorption in experimental periodontitis in mice [70]
DNMTs	AZA	Induction of differentiation of gingival fibroblasts into osteoblasts and induction of ectopic bone formation in mice [120]
		Suppression of *T. denticola*-induced MMP2 activation in PDL cells [29]
	AZA, decitabine	Modulation of inflammatory cytokine production by GECs [121]
	RG108	Prevention of *P. gingivalis*-mediated impairment of GEC barrier function [114]

Although comprehensive analyses of changes in GEC activation upon treatment with HDACi are lacking, an initial study demonstrated that these compounds might enhance expression of *IL8*, *CCL20*, and *hBD2* in GECs infected with *P. gingivalis* or *F. nucleatum* [35]. Because of their antimicrobial activity, induction of human β-defensin-2 (hBD2) and CCL20 by HDACi has been proposed to promote innate immune responses against oral pathogens. This finding is not surprising, given the well-documented ability of these compounds to induce antimicrobial peptides in epithelial cells from different tissues [58]. It should be noted, however, that individual *P. gingivalis* strains are resistant to hBD2 bactericidal activity [59], and direct antimicrobial activity of CCL20 against oral pathogens has not yet been demonstrated. Collectively, these findings highlight the importance of HDAC activity in regulating immune responses at the cellular level, but more comprehensive studies are needed to fully characterize the influences of HDAC inhibitors on global inflammatory mediator output by periodontal tissues. However, experiments on human gingival tissue explants are challenging due to the scarcity of material. Because of that, the recently developed organotypic 3D models comprised of GECs and gingival fibroblasts [60], which mimic many features of the native gingiva, may prove useful for this purpose.

To date, experimental effort has focused primarily on targeting HDAC activity in gingival cells, but a small number of recent studies have analyzed other aspects of chromatin-based epigenetic mechanisms that can be targeted with small molecule inhibitors. The discovery of acetylated histone mimetics that block the interaction between bromodomain-containing BET proteins and acetylated lysine residues on histone proteins has opened a new area for studies of the role of protein acetylation in human diseases [61]. These compounds have been highly effective in suppressing inflammation in myeloid cells [62], synovial fibroblasts [63], and in vivo models of inflammatory diseases [62, 64]. In our recent study, we have shown that inflammatory gene expression is suppressed by the BET inhibitors I-BET151 and JQ1 in gingival fibroblasts and the GEC line TIGK (telomerase-immortalized gingival keratinocyte) [65]. BET inhibitors reduce gingival cell activation induced by *P. gingivalis* and inflammatory cytokines and, importantly, are equally effective in suppressing the expression of inflammatory mediators in GFs derived from healthy donors and periodontitis patients [65]. The cluster of mediators susceptible to inhibition by I-BET151 and JQ1 in gingival fibroblasts and GECs includes IL-6, IL-1β, and CCL2 [65], elevated levels of which are most commonly found in patients with periodontitis [66]. This highlights the potential of BET inhibitors to attenuate crucial mediators of periodontal inflammation.

Although the key roles of histone methylation, in particular H3K4Me3 and H3K27Me3 marks, have been characterized in cellular and animal models of periodontitis [41], the therapeutic potential of compounds that modulate this process has not been evaluated. Gene silencing studies demonstrated that manipulation of enzymes that regulate histone methylation can suppress inflammatory activation and modulate the ability of PDL cells to differentiate into osteoblasts. The silencing of JMJD3 reduces LPS-induced IL-6, IL-8 and IL-12 production [38] and results in diminished alkaline phosphatase activity, indicative of reduced osteogenic potential [40]. SETD1B knockdown results in reduced *IL6* and *IL1B* expression, associated with lower H3K4Me3 levels at *IL6* and *IL1B* promoters [39]. It remains to be tested if pharmacological modulation of histone methylation regulators can recapitulate the effects of gene silencing and suppress pathological inflammatory processes in periodontitis. Of note, the selective JMJD3 inhibitor GSK-J1 displays strong anti-inflammatory activity in macrophages [67].

### Targeting histone PTMs in experimental periodontitis

In vivo, targeting histone acetylation with HDACi or acetylated histone mimetics protects from alveolar bone loss in experimental periodontitis in rodents [68–70] (Table 1). The therapeutic effects of HDACi in *P. gingivalis*-induced periodontitis in mice are dependent on their selectivity: 1179.4b, which targets both class I and class II HDACs, was more potent in suppressing bone destruction than the class I-selective MS-275 (which preferentially targets HDAC1) [69]. Intriguingly, prevention of bone loss in HDACi-treated animals was not associated with reduced immune cell infiltration [69]. These findings indicate that, at least in this model, the amelioration of disease severity by HDACi was unrelated to inflammation. In light of the well-documented ability of HDACi to suppress osteoclastogenesis [71], and recent evidence that these compounds induce osteogenic differentiation of PDL cells [56, 57], the protective effect of 1179.4b may be attributed to the influence of HDACi on alveolar bone regeneration. Indeed, the bone-protective effects of TSA in ligature-induced periodontitis in rats were associated with the ability of HDACi to induce osteogenic differentiation of mesenchymal stem cells [68], though this effect has not been formally demonstrated in vivo.

The observation that HDACi failed to affect inflammation in a murine periodontitis model [69] is surprising and requires independent verification using HDACi with well-characterized pharmacokinetic properties and focusing on immune and microbiological disease parameters. In contrast, treatment of mice with the BET inhibitor JQ1 reduced both alveolar bone resorption and inflammatory cytokine expression in experimental periodontitis [70]. The protective effects of JQ1 were associated with suppressed osteoclast formation [70], consistent with observations in other bone-related pathologies [72]. These results suggest that HDACs and BET proteins may represent attractive targets for host modulation therapy due to their roles in bone resorption. However, it should be noted that increased susceptibility to some bacterial infections has been reported in mice treated with HDACi [73] and BET inhibitors [74]. Therefore, it will be particularly important to determine the influence of these compounds on pathological plaque formation and bacterial clearance in animal models of periodontitis.

## DNA methylation in periodontitis

### Clinical observations: aberrant DNA methylation in samples from periodontitis patients

DNA methylation profiles of selected gene promoters have been investigated in genetic material extracted from human gingival biopsies in numerous studies. The alterations in the promoter methylation status of genes coding for proteins associated with inflammatory tissue responses and for receptors, signaling molecules, and transcription factors that have been observed in patients with periodontitis are summarized in Table 2. It is important to note the discrepancies between the reports: in the case of *IFNG*, *IL6*, *IL8, TNF*, and *TLR2*, the differences in promoter methylation between patients with periodontitis and healthy individuals observed in some studies have not been reproduced in independent analyses [75–86]. Among gene promoters analyzed in more than one study, the results were consistent only in the case of *PTGS2* [77, 84, 85] and *STAT5A* [78, 87], showing increased and decreased methylation in periodontitis patients, respectively. A microarray-based, high-throughput analysis of DNA methylation in healthy donors and patients with periodontitis demonstrated higher variation of changes in genes related to immune responses, and more frequent decreases in their promoter methylation, though specific genes or gene clusters have not been reported [88]. In patients with aggressive periodontitis, reduced levels of DNA methylation in the promoters of *CCL25* and *IL17C* compared to healthy controls have been identified [89]. Curiously, in many genes that are differentially methylated in chronic periodontitis, similar changes in DNA methylation have not been observed in aggressive disease (Table 2).

**Table 2:** Alterations in promoter methylation of selected genes in the gingival tissue from patients with periodontitis

Gene promoter	Study authors	Number of participants (healthy individuals: patients with periodontitis)	Outcome
Genes associated with tissue responses			
IFNG	Zhang et al. [75]	23:12 (+ 12 participants with experimentally induced gingivitis)	↓ (no difference between experimentally induced gingivitis and healthy subjects)
	Viana et al. [76]	16:18	─
	Asa’ad et al. [77]	10:10 (methylation was also assessed 2 and 8 weeks post-therapy)	─ (no change in the course of periodontal therapy)
IL6	Barros and Offenbacher [78]	10:10	↑
	Kobayashi et al. [79]	30:30	─
	Stefani et al. [80]	21:21	─
IL10	Viana et al. [76]	16:18	─
IL17	Barros and Offenbacher [78]	10:10	↑
IL17C	Schulz et al. [89]	10:15 (aggressive periodontitis patients)	↓
CXCL3	Barros and Offenbacher [78]	10:10	↑
	Schulz et al. [89]	10:15 (aggressive periodontitis patients)	─
CXCL5	Barros and Offenbacher [78]	10:10	↑
	Schulz et al. [89]	10:15 (aggressive periodontitis patients)	─
IL8	Barros and Offenbacher [78]	10:10	↓
	Oliveira et al. [81]	41:70 (periodontitis group divided into 30 smokers and 40 non-smokers)	─ (no difference between smokers and non-smokers)
CXCL10	Barros and Offenbacher [78]	10:10	↑
CCL25	Schulz et al. [89]	10:15 (aggressive periodontitis patients)	↓
TNF	Zhang et al. [82]	17:18 (+ 11 participants with experimentally induced gingivitis)	↑ (no difference between experimentally induced gingivitis and healthy subjects)
	Asa’ad et al. [77]	10:10 (methylation was also assessed 2- and 8-weeks after therapy)	─ (no change in the course of periodontal therapy)
PTGS2 (COX2)	Zhang et al. [84]	6:10	↑
	Loo et al. [85]	108:110 (comparison between blood samples from healthy donors and gingival tissue biopsies from patients with periodontitis)	↑
	Asa’ad et al. [77];	10:10 (methylation was also assessed 2- and 8-weeks after therapy)	↑ (periodontal treatment reduced the methylation status to the levels observed in healthy subjects)
Genes coding for receptors, signaling molecules and transcription factors of inflammation-related pathways			
TLR2	de Faria Amormino et al. [86]	20:20	↑
	De Oliveira et al. [83];	11:23 (periodontitis group was divided into 11 smokers and 12 non-smokers)	─ (inconclusive results: mosaic of methylated and unmethylated DNA. Site-specific (restriction enzyme-specific) trend toward increased methylation in periodontitis non-smokers)
	Barros and Offenbacher [78]	10:10	─ (strong trend toward decreased methylation in patients with periodontitis, which did not reach statistical significance)
TLR4	De Oliveira et al. [83];	11:23 (periodontitis group was divided into 11 smokers and 12 non-smokers)	─ (no difference between smokers and non-smokers)
IL4R	Barros and Offenbacher [78]	10:10	↓
	Schulz et al*.* [89]	10:15 (aggressive periodontitis patients)	─
IL6ST	Barros and Offenbacher [78]	10:10	↑
	Schulz et al. [89]	10:15 (aggressive periodontitis patients)	─
TNFRSF18	Barros and Offenbacher [78]	10:10	↑
STAT5A	Barros and Offenbacher [78]	10:10	↓
	Azevedo et al*.* [87]	20:20	↓
TYK2	Barros and Offenbacher [78]	10:10	─
	Schulz et al*.* [89]	10:15 (aggressive periodontitis patients)	─
SOCS1	Planello et al*.* [146]	44:46	↓
SOCS3	Barros and Offenbacher [78]	10:10	↑
RUNX	Barros and Offenbacher [78]	10:10	↑
GATA3	Barros and Offenbacher [78]	10:10	↑
	Schulz et al*.* [89]	10:15 (aggressive periodontitis patients)	─

The presented panel includes genes that are known to be involved in inflammatory events related to periodontitis that are divided into two groups: genes associated with tissue responses and genes coding for receptors, signaling molecules and transcription factors. The analyses have been conducted on genetic material isolated from homogenized human gingival biopsies unless otherwise indicated. The numbers of participants in both groups (healthy individuals: patients with periodontitis) are shown in parentheses for each study. ↓—decreased methylation in patients with periodontitis compared to healthy controls; ↑—increased methylation in patients with periodontitis compared to healthy controls; ─—no difference between groups

Alterations in the DNA methylation profiles in periodontitis are not restricted to the site of inflammation. Changes in gene promoter methylation status have also been shown in blood samples (Table 3). Increased methylation of the *TNF* promoter region [90] and decreased *IL6* promoter methylation [91] have been observed in the peripheral blood from patients with periodontitis, though the latter observation has not been confirmed in an independent study [79]. The levels of *IL6* promoter methylation in the blood are significantly higher in comparison to the gingival tissue [79], and similar differences in global 5hmC levels between tissue and blood have been observed [92]. Moreover, epigenome-wide association scans (EWAS) performed on blood samples in a twin study identified a number of differentially methylated genes related to self-reported periodontal traits: gingival bleeding and tooth motility [93]. Eight of these genes, including *CXCL1*, *IL1B*, *IL6ST*, and *CD44*, were consistently differentially methylated in participants with periodontitis-related traits [93].

**Table 3:** Alterations in promoter methylation of selected genes in the blood and buccal mucosa from patients with periodontitis

Gene promoter	Study authors	Number of participants (healthy individuals:patients with periodontitis)	Outcome
Blood			
TNF	Kojima et al*.* [90]	30:30 (+ 30 patients with RA)	↑ (the same effect observed in RA, but in higher number of CpG sites)
	Kobayashi et al. [79]	30:30	─
IL6	Ishida et al. [91]	30:30 (+ 30 patients with RA)	↓ (the same effect observed in RA)
	Kobayashi et al. [79]	30:30	─
VDR	Kurushima et al. [93]	EWAS twin study, correlation with 2 different periodontal traits was analyzed separately in participants from TwinsUK registry (83% monozygotic and 10% dizygotic twins) Gingival bleeding (528 participants: 259 negative vs 269 positive) Tooth mobility (492 participants: 371 negative vs 121 positive) There was an overlap of 474 participants between the analyses	↕
IL6ST			
TMCO6			
IL1RN			
CD44			
IL1B			
WHAMM			
CXCL1			
Buccal mucosa			
IL8	Oliveira et al. [81]	41:70 (periodontitis group was divided into 30 smokers and 40 non-smokers)	↓ (no difference between smokers and non-smokers)
	Andia et al. [94]	37:37 (aggressive periodontitis patients)	↓
SOCS1	Baptista et al*.* [95]	30:30 (aggressive periodontitis patients)	↑
VDR	Kurushima et al. [93]	EWAS twin study, correlation with 2 Different periodontal traits were analyzed separately: Gingival bleeding (43 participants: 18 negative vs 25 positive; 20 monozygotic twins, 16 dizygotic twins and 7 singletons) Tooth mobility (41 participants: 29 negative vs 12 positive; 20 monozygotic twins, 12 dizygotic twins and 9 singletons) There was an overlap of 40 participants between the analyses	↕
IL6ST			
TMCO6			
IL1RN			
CD44			
IL1B			
WHAMM			
CXCL1			
MMP13			
MED24			
CCR1			
MMP3			
TLR4			
IL6			
IL10			
SNORD124			

The numbers of participants in both groups (healthy individuals: patients with periodontitis) are shown in parentheses for each study. ↓—decreased methylation in patients with periodontitis compared to healthy controls; ↑—increased methylation in patients with periodontitis compared with healthy controls; ─—no difference between groups; ↕—differential methylation (effect direction not stated)

Changes in site-specific DNA methylation have also been reported in the buccal mucosa from patients with periodontitis (Table 3). Lower methylation levels of the *IL8* promoter region have been observed in buccal epithelial cells from periodontitis patients compared to healthy controls, though they were not accompanied by similar differences in gingival biopsies [81]. A decrease in *IL8* promoter methylation has also been shown in buccal epithelial cells from patients with aggressive periodontitis [94], whereas methylation levels of the *SOCS1* promoter and the long interspersed nucleotide elements (LINE1 elements) have been elevated [95]. LINE1 elements are repetitive DNA sequences known to undergo heavy methylation. Because of that, they are commonly used as a surrogate marker for global DNA methylation.[96]. Interestingly, a similar profile of associations between DNA methylation and periodontal traits across blood and the buccal tissue has been observed for a small number of genes in the EWAS study [93]. Since the microbiome of the buccal mucosa is altered in patients with periodontal disease [97] and the tissue is easily accessible, understanding epigenetic changes within this site could potentially be useful for diagnostic purposes.

In addition to gene-specific methylation studies, expression of DNA methyltransferases and demethylating enzymes, as well as global DNA methylation levels have been assessed in gingival tissues. Similar transcript levels of *DNMT1* and *DNMT3a* are expressed in gingival biopsies from periodontitis patients and healthy controls [98]. Consistently, Larsson et al. found no differences in DNMT1 expression between periodontitis and gingivitis patients. Also, there was no difference in global 5mC or 5hmC levels assessed by immunohistochemistry. In contrast, increased numbers of TET2-positive cells have been found in samples from periodontitis patients [92], though they were not associated with similar differences in *TET2* mRNA expression in the total gingival tissue, suggesting that this effect may be secondary to cellular heterogeneity of the sample [92]. Collectively, these results indicate that alterations in the promoter-specific methylation profiles observed in periodontitis are not caused by global DNA hyper- or hypomethylation, or by alterations in the expression of enzymes that regulate this process that could be detected in total gingival tissue samples. Instead, they are most likely driven locally by exposure to bacterial factors or the inflammatory microenvironment and are cell type-specific.

The biological relevance of the reported changes in DNA methylation in periodontitis is supported by studies of their relationship with gene expression. Negative correlations between the levels of DNA methylation at gene promoter regions and gene expression have been found for *TNF* [82], *PTGS2* [84], *TLR2* [86], and *IFNG* [75] in gingival biopsies, and for *IL8* in oral epithelial cells [81]. In blood samples, promoter methylation of *TNF* [90], but not *IL6* [91], inversely correlates with serum protein levels. An overall negative correlation between DNA methylation levels within gene promoters and gene expression in the gingival tissue has also been confirmed in a high-throughput analysis [88]. Correlations between DNA methylation and gene expression, however essential, provide limited information about the causative relationship between the two in the absence of functional studies. Furthermore, DNA methylation studies performed to date have predominantly focused on effector molecules, such as cytokines and chemokines. Although important, they are not sufficient for identification of specific gene networks and early signaling events that are dysregulated in the inflamed gingival tissue through epigenetic mechanisms and may represent attractive targets for therapeutic intervention. Thus far, few genes coding for receptors, signaling molecules and transcription factors have been analyzed in relatively small patient cohorts (Table 2). Well-designed high throughput epigenomic and transcriptomic studies are therefore needed to identify upstream signaling pathway components that are dysregulated in periodontitis through changes in DNA methylation (or other epigenetic mechanisms).

Potential relationships between clinical diagnostic parameters of periodontitis patients and DNA methylation of selected gene promoters have been assessed in two independent studies, identifying correlations of DNA methylation status with periodontal probing depth (PPD) for two promoter regions (positive correlation for *TLR2* [86] and negative for *IL-6* [79]). In contrast, no correlation was observed between gene methylation status and clinical attachment loss (CAL) as well as % sites with bleeding on probing (BOP) [79, 86]. De Faria Amormino et al. [86] have also found a positive correlation between methylation of the *TLR2* promoter and the number of inflammatory cells in the periodontal tissue from both healthy donors and patients with periodontitis. However, this correlation may be partly explained by differences in the cellular composition of healthy and inflamed tissue, especially the influx of leukocytes during inflammation [86]. Similarly, alterations in *GATA3* methylation observed in periodontitis may simply reflect the accumulation of a specific lymphocyte population [78]. Increased numbers of inflammatory cells are associated with spatial differences in gene expression within periodontitis-affected gingival tissue [99]. Similar spatial analysis of DNA methylation combined with gene expression profiling could immensely contribute to a better understanding of the effects observed in studies of whole tissue samples.

Demethylation of specific CpG sites has been observed as a consequence of transcription factor binding during inflammatory responses, leading to the heterogenic methylation pattern within certain promoters [100, 101]. The application of different methylation-sensitive enzymes revealed the heterogeneity of DNA methylation within the *TLR2* promoter in the gingival tissue [83]. In the same study, a trend toward increased methylation in periodontitis patients has been shown for a specific CpG site, but not for the whole *TLR2* promoter [83]. Similar heterogeneity of DNA methylation within individual promoters and site-specific differences between healthy donors and patients with periodontitis have been observed for the *TNF* [82] and *IFNG* [75] genes analyzed by pyrosequencing. Such changes in DNA methylation restricted to specific CpG sites may be biologically relevant because, in many cases, the methylation status of individual transcription factor-binding sites is crucial for the regulation of gene expression [102]. However, the application of techniques assessing only site-specific DNA methylation, such as bisulfite-conversion or digestion-based PCR methods, may partly explain divergent results regarding promoter methylation of the same genes analyzed in independent studies [103]. Differences in patient inclusion criteria and technical aspects of sample collection and processing may also contribute to the observed discrepancies.

Finally, little is known about the impact of potential confounding factors on the reported changes in DNA methylation in periodontitis patients. Among them, the cellular heterogeneity of tissue biopsies (as well as whole blood samples) may be the most important confounder affecting the outcome in cross-sectional studies [104]. It is also important to note that changes in DNA methylation in the total gingival tissue may not reflect differences in individual cell populations. Barros et al. [78] isolated GECs from gingival tissue biopsies using the laser capture microdissection method and found a cluster of inflammatory genes with increased methylation levels in cells from periodontitis patients (*TYK2*, *IL17C, IL12B, CCL25, CXCL14, IL4R*). Comparing this cluster to the genes with differential methylation profiles in periodontitis-affected gingival tissue from the same patients, a similar effect was observed only in the case of *CXCL14* [78]. Genetic variation, age, and environmental risk factors that contribute to periodontitis susceptibility may also influence DNA methylation independent of disease-related processes [104]. Smoking is one of the most important, preventable risk-factor for periodontal disease [105], which directly influences DNA methylation [106]. Promoter-specific changes in DNA methylation in smokers and non-smokers with periodontitis have been compared in two studies, finding little or no differences between the groups (Table 2) [81, 83]. Ageing-related changes in DNA methylation correlate with increased mortality in a number of age-dependent disorders as well as activation of pro-inflammatory pathways [107]. These changes are also influenced by obesity and alcohol consumption [108], which are emerging risk factors for periodontitis [109, 110], but have not been considered in the studies available to date.

### Changes of DNA methylation profiles in resident cells of the periodontium

**Fig. 2:**
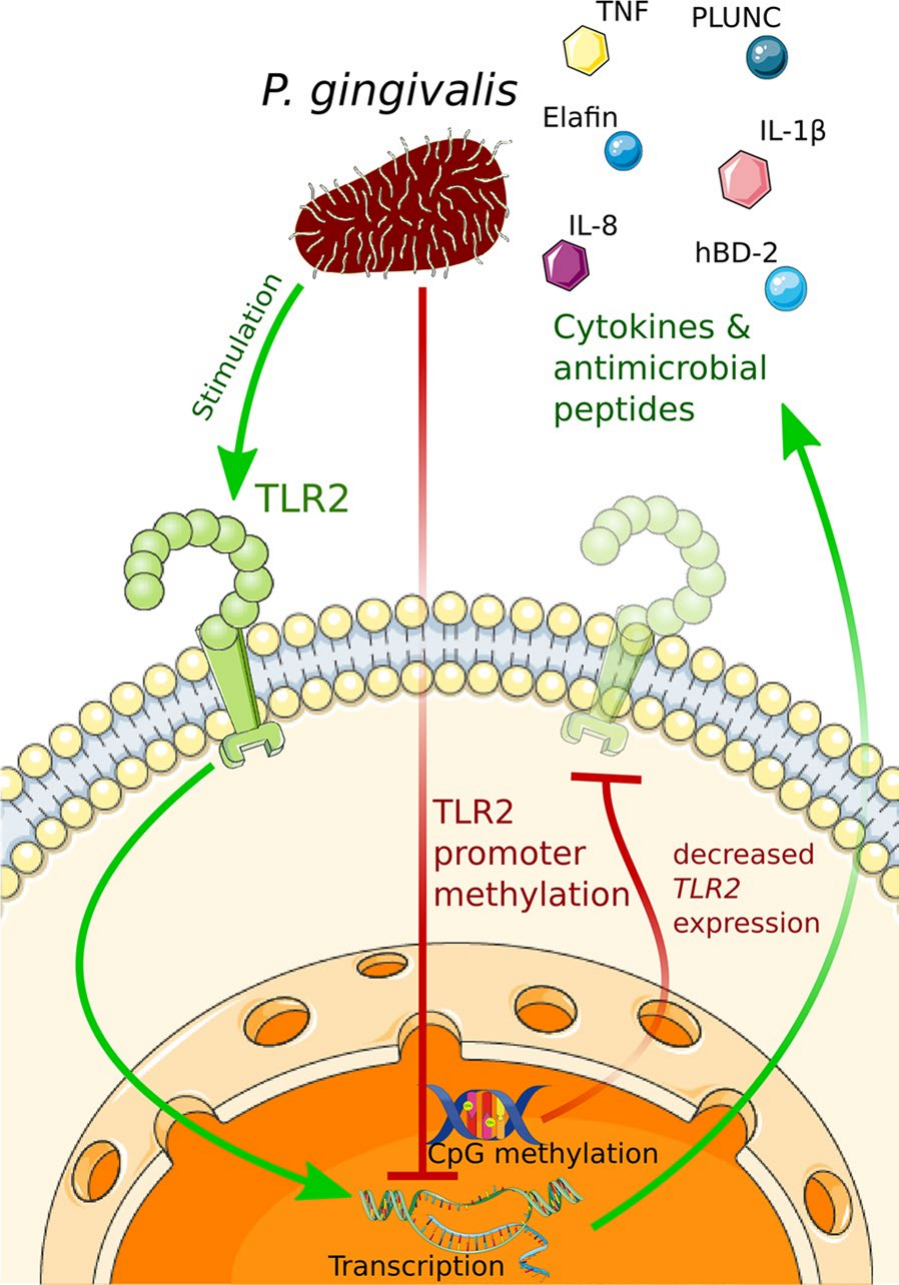
Model of epigenetic regulation of TLR2 expression in GECs. Cell responses to *P. gingivalis* are predominantly mediated by engagement of TLR2. Under physiological conditions, *P. gingivalis*-induced activation of TLR2 stimulates the production of inflammatory cytokines, chemokines and antimicrobial peptides that promote pathogen elimination by the immune system. Chronic exposure to *P. gingivalis,* through an unknown mechanism, induces increased *TLR2* promoter methylation in GECs, which was also observed in cells from a subgroup of periodontitis patients [111]. *TLR2* promoter hypermethylation is associated with reduced TLR2 expression and diminished production of inflammatory mediators and antimicrobial peptides normally induced by the pathogen [111]. This figure was created using images from Servier Medical Art (http://smart.servier.com). Servier Medical Art by Servier is licensed under a Creative Commons Attribution 3.0 Unported License

Analyses of DNA methylation at individual gene promoters indicate that oral pathogens and inflammatory stimuli tend to promote gene-specific hypermethylation in gingival cells. In the most comprehensive study performed to date, Benakanakere et al. [111] demonstrated that blunted inflammatory responses to *P. gingivalis* observed in GECs from a subset of periodontitis patients are associated with diminished TLR2 expression and increased methylation of the *TLR2* promoter region. Consistently, chronic infection with *P. gingivalis* induces *TLR2* promoter hypermethylation both in GEC cultures and the murine gingiva [111]. Since TLR2 is a central regulator of immune responses to *P. gingivalis* and is necessary for the development of pathological inflammation in murine models of periodontitis [112, 113], DNA methylation may be a crucial component of a mechanism controlling GEC responses to this periopathogen (Fig. 2). *P. gingivalis* infection of GECs also leads to increased methylation and reduced expression of genes coding for the components of the cell–cell junction complexes (*CDH1*, *PKP2*, and *TJP1*), which results in functional impairment of the epithelial barrier [114]. Chronic stimulation of PDL cells with *P. gingivalis* LPS promotes hypermethylation of several genes encoding ECM components, including *FANK1, COL4A1-A2, COL12A1, COL15A1, LAMA5, LAMB1, MMP25, POMT1,* and *EMILIN3,* that is associated with reduced expression levels of these genes [115]. Preliminary analyses performed on a small number of gingival fibroblast lines also indicate differential methylation of individual gene promoters (*CD40*, *IL8*, *TNFRSF-10C*, and *MMP13*) upon stimulation with IL-1β or PGE2, with increased methylation observed at the majority of the analyzed CpG sites [116].

A number of studies conducted on isolated gingival and periodontal cell populations have evaluated the expression of DNMTs and/or global changes in DNA methylation in response to specific factors involved in periodontitis pathogenesis. Decreased expression of DNMT1 has been observed in primary GECs after infection with *P. gingivalis* or *F. nucleatum* [35]. Similarly, treatment with *P. gingivalis* LPS leads to the downregulation of DNMT1 in PDL stem cells [117] and the HaCat keratinocyte cell line, but not in gingival fibroblasts [98]. *DNMT3a* mRNA expression is also reduced in HaCat cells exposed to *P. gingivalis* LPS [98]. In gingival fibroblasts, DNMTs are also regulated by inflammatory mediators that play a central role in periodontitis pathogenesis: IL-1β upregulates DNMT1 and downregulates DNMT3a expression, whereas exposure to PGE2 leads to downregulation of both DNMT1 and DNMT3a [116]. Interestingly, the relationship between DNMT expression and global DNA methylation is not straightforward as increased global 5mC levels have been observed in gingival fibroblasts after stimulation with either IL-1β or PGE2, with a stronger effect of the latter despite downregulation of both DNMTs [116].

Cytidine structural analogs 5-azacytidine (AZA) and 5-aza-2′-deoxycytidine (decitabine) can be used to specifically inhibit DNMT1 [118], though they may also influence other targets [119]. Treatment of gingival fibroblasts with AZA results in increased expression of osteogenic lineage markers RUNX and ALP associated with demethylation of their promoter regions [120]. Furthermore, pretreatment with AZA before culture in the presence of bone morphogenetic protein-2 (BMP2) promotes differentiation of gingival fibroblasts into functional osteoblasts, which may represent a novel therapeutic strategy for bone regeneration [120]. DNMT1 inhibition with AZA also suppresses *T. denticola*-induced upregulation of MMP2 and components of its activating complex (MT1-MMP and TIMP-2) in PDL cells, though it has not been tested whether this is caused by changes in MMP2 promoter methylation status [29]. In GECs, inhibition of DNMT1 modulates production of inflammatory mediators induced by oral bacteria in a complex manner: pretreatment with AZA enhanced *P. gingivalis*-induced expression of CCL20, hBD2 [35], and IL-1α, and suppressed induction of IL-6 and CXCL1 by *P. gingivalis*, or *F. nucleatum* [121]. A similar pattern of changes in gene expression has been observed in GECs treated with decitabine prior to infection with *P. gingivalis* but, surprisingly, not with *F. nucleatum* [121]. However, in these studies, GECs were exposed to DNMT1 inhibitors for a short period before infection (4 h) [35, 121], which may be insufficient to induce DNA hypomethylation. Finally, a recent study demonstrated that pretreatment of GECs with non-nucleoside DNMT inhibitors (RG-108, EGCG, or curcumin) could reverse the effects of *P. gingivalis* on methylation and expression of a cluster of genes encoding components of cell–cell junction complexes, as well as functional impairment of the gingival epithelial barrier [114].

Activation of gingival cells is also regulated by proteins involved in DNA demethylation, mainly TET enzymes. In gingival fibroblasts, stimulation with IL-1β or PGE2 results in decreased transcription of the *TET1* gene but increased 5hmC levels [116]. Elevated 5hmC levels might be caused by diminished TET1 expression; since the enzyme has higher catalytic activity toward 5mC than its oxidized derivatives, its diminished expression may be insufficient to further oxidize 5hmC, ultimately leading to accumulation of this DNA modification [122]. However, it should be noted that the increases in 5hmC levels observed in gingival fibroblasts upon stimulation are accompanied by simultaneous decreases in 5mC levels [116]. It is yet to be assessed how pro-inflammatory stimulation modulates the expression of other TET enzymes in these cells, in particular TET2, which plays a substantial role in innate immune responses of myeloid cells [16]. Since the TET enzymes have been shown to protect specific CpG sites from methylation [123], downregulation of TET1 in gingival fibroblasts upon inflammatory stimulation may also partially explain DNA hypermethylation observed despite decreased DNMT1 and DNMT3a expression [116]. TET1 is also involved in the differentiation of THP-1 cells into M1 macrophages after co-stimulation with *P. gingivalis* LPS and IFN-γ [124]. Similarly, TET2 silencing results in reduced cytokine expression in dental pulp cells after LPS stimulation, associated with decreased *MYD88* gene promoter hydroxymethylation levels and diminished NF-κB signaling [125]. As these observations are based on gene silencing approaches, it should be noted that the TET enzymes can also regulate immune responses through mechanisms independent of DNA demethylation, including HDAC2 recruitment [126]. Moreover, studies in dendritic cells suggest that DNA demethylation might not be the cause of induced gene expression, but rather its downstream consequence [101].

### In vivo studies of DNA methylation in periodontitis

Although animal models of periodontitis are an excellent tool for spatiotemporal analysis of changes observed in patient-derived samples, they have not been widely used to study DNA methylation. Immunostaining of DNMT3b has been recently used as a marker of de novo DNA methylation to analyze the relationship between local and systemic infection in murine periodontitis models [127]. Palioto et al. [127] demonstrated that systemic microbial challenge with *P. gingivalis* by oral gavage, but not local challenge induced by ligature placement, increases DNMT3b levels in the gingival tissue, especially on the alveolar bone surface. Interestingly, similar changes in DNMT3b expression have been noted in the gut epithelium, suggesting that systemic effects of the bacterial challenge may be mediated through epigenetic mechanisms [127]. Murine models have also been used to confirm or complement in vitro studies, demonstrating increased *TLR2* promoter methylation in the gingival tissue of mice challenged with *P. gingivalis* [111] and induction of ectopic bone formation by the DNMT inhibitor AZA [120].

Early stages of periodontitis development can also be studied in humans in experimental gingivitis that is induced by restraining from oral hygiene [128]. In this model, *IFNG* mRNA expression was even higher than in periodontitis patients, but returned to the levels seen in periodontal health after resolution of inflammation [75]. Curiously, this increase in gene expression in individuals with experimental gingivitis was not accompanied by decreased *IFNG* promoter methylation, which was noted in patients with periodontitis assessed in parallel [75]. Similarly, increased *TNF* promoter methylation, found in periodontitis patients, was not observed in experimental gingivitis, but in this case, decreased mRNA levels were observed only in patients with periodontal disease [82]. As gingivitis, in contrast to periodontitis, is a reversible form of inflammation, these results could indicate that alterations in DNA methylation can contribute to failure of inflammation resolution that is an important aspect of periodontitis pathogenesis [129]. Notably, pathological changes in DNA methylation can be reversed following periodontal treatment. Asa’ad et al. [77] demonstrated that in patients with periodontitis, the methylation status of the *PTGS2* promoter, which was differentially methylated compared to healthy individuals, returned to the levels associated with periodontal health after conventional periodontal therapy (Table 2).

## Epigenetic changes as potential diagnostic and therapeutic targets in periodontitis

In recent years, specific DNA methylation signatures in blood samples have been thoroughly investigated as biomarkers of certain types of cancer, and clinical assays detecting them are already commercially available [130], paving the way for the use of DNA methylation in the diagnostics of disorders with underlying epigenetic mechanisms [131]. Alterations in DNA methylation status have been linked to inflammation and are currently evaluated as potential biomarkers in multiple diseases, including Crohn’s disease [132] and lupus [133], and differential DNA methylation is associated with response to treatment in RA [134]. In periodontitis patients, both promoter-specific analyses [79, 90, 91] and EWAS [93] have identified altered DNA methylation signatures in blood samples (Table 3), raising the possibility that some of them, upon validation in larger patient cohorts, may be used for diagnostic purposes. However, their relationships with disease susceptibility, progression, or severity are yet to be assessed, which will be necessary before considering their applicability as disease biomarkers. The buccal mucosa is another easily accessible tissue which could be used for diagnostic purposes and altered DNA methylation patterns have been observed in buccal epithelial cells from patients with chronic [81] and aggressive [94, 95] forms of the disease. Since the buccal tissue more closely interacts with the pathological oral microbiota compared to peripheral blood samples [97], it could provide more relevant information about disease pathogenesis.

Identification of confounding factors that may affect potential epigenetic markers will be crucial for determining their applicability in diagnostic tests and proper control group selection. It is important to note that DNA methylation patterns differ between samples from gingival biopsies and the buccal mucosa [81] or blood at the specific CpG sites [79] and globally [92]. It is currently unknown whether these discrepancies are related to the disease pathobiology or are secondary to differences in cellular sample composition, which are a well-known confounder in epigenetic studies [104]. Other factors known to affect DNA methylation patterns, such as genetic polymorphisms, age [104], and environmental risk factors [135] also need to considered.

The successful introduction of epigenetic drugs into clinical practice in oncology and the discovery of anti-inflammatory properties of HDAC and BET inhibitors have highlighted the potential for targeting epigenetic modifications for the treatment of multiple diseases. The reported initial clinical efficacy of HDACi in patients with graft-versus-host disease [136] and systemic-onset juvenile idiopathic arthritis [137], in combination with an acceptable profile of adverse events observed in these trials, have further stimulated interest in targeting histone acetylation in immune-mediated inflammatory diseases. The available in vitro and in vivo observations support the idea that targeting HDACs or BET proteins may be beneficial in periodontitis by suppressing excessive inflammation and restoring bone homeostasis (Table 1).

However, attempts to target epigenetic modifications in patients with periodontal disease need to proceed with caution. First, evidence from clinical trials in cancer patients indicates that broad-spectrum HDACi have a number of dose-limiting toxicities, including thrombocytopenia, neutropenia, fatigue, and diarrhea [138]. Such a profile of undesirable adverse effects can be tolerated in patients with life-threatening cancer but will not be acceptable in milder conditions. It is, however, important to note that HDACi exert their anti-inflammatory properties at significantly lower concentrations compared to those required to induce apoptosis of tumor cells [139]. Therefore, the doses required to achieve efficacy in patients with inflammatory conditions will likely have a more favorable safety profile. Similarly, it is expected that targeting individual HDACs with selective HDACi will result in reduced toxicity while achieving anti-inflammatory effects similar to pan-HDACi. In vitro studies indicate that HDAC3 inhibition may be sufficient to suppress inflammatory activation [53, 54], though HDAC3-selective compounds have yet to enter clinical trials. Second, while the reduction of excessive and non-resolving inflammation is the primary goal of host modulation therapy, it should not impair host responses to oral and other pathogens [1]. The available studies have identified several pathogen- and cell type-specific effects of HDACi on antimicrobial responses: while HDACi-mediated induction of antimicrobial peptides by epithelial cells could facilitate pathogen elimination, suppression of macrophage and dendritic cell immune responses may increase susceptibility to specific bacterial or fungal infections [140]. Similar concerns need to be addressed regarding the potential application of BET inhibitors in periodontitis. These compounds have progressed into clinical trials in cancer [141], but have not been evaluated in patients with any inflammatory conditions, and their effects on host–pathogen interactions remain to be characterized.

Comprehensive in vivo studies analyzing long-term effects of HDACi or BET inhibitor treatment on host responses to the dysbiotic oral microflora (as well as other pathogens not related to the oral microbiota) are therefore necessary to determine the potential consequences of epigenetic therapies on the oral health and to identify the optimal mode of drug administration. The available literature does not provide definitive indications whether epigenetic changes should be targeted systemically or locally at the site of inflammation. This will ultimately depend on the relative contributions of dysregulated epigenetic mechanisms to the pathological activation of different cell types that drive chronic inflammation in periodontitis. These cell type-specific effects may be impossible to address in studies of patients with established disease and will require a well-designed animal model and longitudinal studies of individuals at risk of developing periodontitis. This approach will allow for understating the complex relationships between systemic epigenetic changes in circulating immune cells and alterations that occur locally in resident cells of the periodontium at different stages of disease development and progression.

## Summary and future perspectives

Strong experimental evidence has accumulated in the last decade that epigenetic regulatory mechanisms are perturbed in periodontitis. While alterations in DNA methylation profiles of genes involved in disease pathogenesis may represent potential candidates for disease biomarkers, proteins that regulate posttranslational histone modifications, in particular acetylation, have emerged as attractive targets for novel host modulation therapies. However, several gaps in our understanding of epigenetic regulation in periodontitis need to be filled before these early findings could be translated into the clinical practice. Still, little is known about the stability of changes in epigenetic marks over time and their long-term effects on gingival cell responses to inflammatory and infectious cues. Studies of gingival fibroblasts from patients with periodontitis indicate that these cells maintain their activated phenotype in vitro and are more active in response to *P. gingivalis* infection [142, 143]. Though the molecular mechanisms underlying this imprinted activation have not been identified, a possibility that it is mediated by alterations in the epigenetic landscape should be verified experimentally. Supported by the discovery of epigenetically-driven “innate immune memory” in macrophages [144], the idea that bacterial infection can induce long-lasting effects in host cells has recently gained significant interest [145]. It is thus far unknown whether this kind of memory exists in gingival cells exposed to oral pathogens and if these changes render patients more susceptible to alterations in the composition of oral microflora.

The rapid development of genomic, epigenomic and transcriptomic methods has allowed for the identification of causal relationships between defects in epigenetic regulation and specific pathological mechanisms in many diseases [10]. These approaches should be used in integrated basic and clinical studies to create a comprehensive map of epigenetic changes in periodontitis and identify cell types where these changes occur. Notably, in vitro studies have predominantly focused on resident cells of periodontal tissues, but little is known about the dysregulation of epigenetic mechanisms in immune cells that infiltrate the inflamed gingiva. Global analyses of the epigenetic landscape need to be accompanied by functional studies delineating the detailed roles of individual epigenetic enzymes in periodontitis development and progression. These studies are necessary to fully comprehend the cellular processes underlying epigenetic alterations observed in patients with periodontitis, their pathophysiological consequences as well as specific environmental factors influencing them. Future research should also improve our understanding of the molecular mechanisms responsible for the anti-inflammatory and bone-protective effects of epigenetic drugs observed in cellular and animal models of periodontitis. The integration of experimental evidence from high-throughput epigenomic and functional studies will facilitate hypothesis-driven decisions regarding the suitability of these compounds, in particular HDACi and BET inhibitors, in the treatment of periodontal disease.

## Data Availability

Not applicable.

## Abbreviations

AZA: 5-Azacytidine
BET: Bromodomain and extra-terminal
DNMT: DNA methyltransferase
EWAS: Epigenome-wide association scan
GEC: Gingival epithelial cell
HAT: Histone acetyltransferase
HDAC: Histone deacetylase
HDACi: Histone deacetylase inhibitor
IL: Interleukin
MMP: Matrix metalloproteinase
PDL: Periodontal ligament
PGE2: Prostaglandin E2
PTM: Post-translational modification
RA: Rheumatoid arthritis
SAHA: Suberoylanilide hydroxamic acid
SCFA: Short-chain fatty acid
TET: Ten-eleven translocation
TNF: Tumor necrosis factor
